# Epigenetic regulatory elements associate with specific histone modifications to prevent silencing of telomeric genes

**DOI:** 10.1093/nar/gkt880

**Published:** 2013-09-25

**Authors:** Stefano Majocchi, Elena Aritonovska, Nicolas Mermod

**Affiliations:** Laboratory of Molecular Biotechnology, Center for Biotechnology UNIL-EPFL, University of Lausanne, 1015 Lausanne, Switzerland

## Abstract

In eukaryotic cells, transgene expression levels may be limited by an unfavourable chromatin structure at the integration site. Epigenetic regulators are DNA sequences which may protect transgenes from such position effect. We evaluated different epigenetic regulators for their ability to protect transgene expression at telomeres, which are commonly associated to low or inconsistent expression because of their repressive chromatin environment. Although to variable extents, matrix attachment regions (MARs), ubiquitous chromatin opening element (UCOE) and the chicken cHS4 insulator acted as barrier elements, protecting a telomeric-distal transgene from silencing. MARs also increased the probability of silent gene reactivation in time-course experiments. Additionally, all MARs improved the level of expression in non-silenced cells, unlike other elements. MARs were associated to histone marks usually linked to actively expressed genes, especially acetylation of histone H3 and H4, suggesting that they may prevent the spread of silencing chromatin by imposing acetylation marks on nearby nucleosomes. Alternatively, an UCOE was found to act by preventing deposition of repressive chromatin marks. We conclude that epigenetic DNA elements used to enhance and stabilize transgene expression all have specific epigenetic signature that might be at the basis of their mode of action.

## INTRODUCTION

In the nucleus of eukaryotic cells, linear chromosomal DNA associates with histones and other proteins to form chromatin. Besides packaging DNA into a smaller volume, chromatin also serves as a mechanism to control DNA expression and replication (1). Numerous factors, including histone modifications, incorporation of histone variants and DNA methylation, affect the chromatin structure and therefore the accessibility of DNA to the transcription and replication machineries. The histone tails can be decorated with a number of modifications. Some of them, such as acetylation of histone H3 and H4 or di/trimethylation of H3K4, are typically associated with active transcription and are therefore referred to as euchromatin modifications. On the other hand, modifications such as trimethylation of H3K9, H3K27 or H4K20 are commonly mapped on inactive genomic regions and termed heterochromatin modifications (1).

Telomeres are regions of highly repetitive DNA that protect the ends of linear chromosomes from DNA repair or recombination (2). Generally, mammalian telomeres are characterized by both hypoacetylation and hypermethylation of selected histone lysines. For instance, trimethylated lysine 9 of histone H3 (H3K9me3) and H4K20me3, which are commonly found in constitutive heterochromatin, are highly enriched at telomeres (3). HP1 proteins, which display high affinity to H3K9me3, are also enriched at telomeres, where they may contribute to further compaction of telomeric and subtelomeric regions (4). Nevertheless, active histone modifications such as H2BK5me1 and H3K4me3 were also found to be enriched at telomeres (5), possibly reflecting the fact that human and mouse telomeres are actively transcribed by RNA polymerase II into long non-coding RNAs termed TERRA (6). Therefore, and contrary to what was believed in the past, the current opinion is that telomeric chromatin is not typical constitutive heterochromatin, although heterochromatic features prevail in the epigenetic landscape of telomeres (7).

The subtelomeric DNA is heavily methylated at CpG dinucleotides, and inactivation of DNA methyltransferases from mouse embryonic stem cells results in telomeric instability (8). Similarly, in the absence of key histone methyltransferases such as SUV39H or SUV420, which are responsible of the trimethylation of H3K9 and H4K20 respectively, the prevailing heterochromatic environment of telomeres is lost, with inauspicious consequence for telomere integrity (9,10). Telomere length is also an important factor for the maintenance of their epigenetic landscape, as a shorter length of the repeated DNA sequences leads to a decrease of heterochromatic marks such as H3K9 and H4K20 trimethylations and to an increase of histone acetylation (11). Recently, additional modifications were found to be associated with subtelomeric sequences, including H3K27me2/3 and H3R2me1 (5).

The particular chromatin environment of telomeres leads to a chromatin-mediated silencing of telomeric-proximal endogenous genes at their native location, as well as that of transgenes integrated at telomeric *loci*. This phenomenon, termed the telomere position effect (TPE), occurs in both lower and higher eukaryotes (12–15). Unlike yeasts where TPE has been associated with the combined actions of Sir proteins which spread from the telomeres along the chromosome (16), the mechanism of mammalian TPE is not fully understood as yet, but it is known to involve SIRT6, a histone deacetylase member of the Sir2 family which specifically targets acetylated H3K9 and H3K56 (17,18). For instance, depletion of SIRT6 was shown to abrogate TPE in HeLa cells by disrupting the prevailing heterochromatic environment of telomeres (19). In addition, telomeric silencing has also been associated with the deposition of heterochromatic marks, such as the methylation of CpG islands at human subtelomeric regions by the DNA methyltransferase 3b (20).

Upon transgene spontaneous integration into the host genome as in stable transfections, both nearby endogenous regulatory elements and the chromatin structure at the genomic *locus* of integration may affect transgene expression, resulting in a position effect which often causes limited level of transgene expression (21). However, various epigenetic regulators that may partially overcome the position effect have been identified in different eukaryotic systems, and some have been successfully used in the expression of recombinant proteins in cultured cells or in gene therapy models (22). Among these elements, insulators or barrier elements have been proposed to partition the genome into discrete chromatin domains (23). An insulator may have enhancer-blocking activity—hence interfering with the enhancer-promoter communication when interposed between them (24)—and/or barrier activity, therefore preventing the spread of repressive heterochromatin over adjacent euchromatin domains (25). Consequently, insulators were shown to confer stability to the transgene expression overtime (26) and they are seen as promising tools to increase the safety of gene therapy vectors (27). Numerous insulators have been identified in different species, but the most extensively studied element is the chicken β-globin 5′ hypersensitive site 4 (cHS4), a potent insulator which was shown to combine both enhancer-blocking and barrier activities (28).

Matrix attachment regions (MARs) and scaffold attachment regions are A + T rich DNA of variable length that were originally found to bind the nuclear matrix or scaffold, a poorly understood protein structure within the cell nucleus (29). They are thought to organize eukaryotic chromatin into distinct regulatory domains by the formation of 50–200 kb structural loops. It is estimated that more than 50 000 MARs may be present in mammalian genomes (30). Although little sequence homology exists among MARs sequences besides an enrichment for AT bases, they must have an evolutionary conservation since MAR elements from one species are functional in another (31). The chicken lysozyme MAR was one of the first MARs to be shown to increase and sustain transgene expression (32,33). Genome-wide studies resulted in the identification of new, more potent MARs, including the human MAR 1-68 and X-29 (30) and the murine MAR S4 (34). Addition of a MAR to an expression vector has three consequences on the resulting stable cell populations: (i) the number of stably expressing cell clones increases; (ii) expression of the transgene is enhanced and stabilized upon long-term cell culturing; (iii) the variability in gene expression within a polyclonal cell population is reduced (33,35). It was proposed that gene expression may be increased by the interaction of MAR elements and endogenous enhancers, or that an active chromatin configuration may be generated upon association to the nuclear matrix. It was also suggested that the AT-rich core may destabilize the double helix and thus promote transcription initiation (31), but the question of how MARs enhance gene expression has yet to be fully answered.

STabilizing Anti-Repressor (STAR) elements are genetic elements proposed to counteract chromatin-associated repression effects, thereby leading to high and stable expression of transgenes (36). They were identified in a genetic screening where only cells containing genetic elements capable of blocking the spreading of heterochromatin-related proteins such as HP1 could survive. STAR elements 7 and 40 were among the most effective members of this family. They form a novel class of genomic DNA elements which are highly conserved between human and mouse, whose size is smaller than MARs, and that can be combined with high-stringency selection system to rapidly create highly protein-productive mammalian cell lines (22).

Ubiquitous chromatin opening elements (UCOEs) are regulatory elements derived from promoter-containing CpG islands of ubiquitously expressed housekeeping genes (37). It was proposed that regulatory elements from such promoters possess a chromatin-remodelling function allowing the maintenance of chromatin in a permissive configuration (38), resulting in high and consistent expression of genes in their proximity (37,39). Although originally relatively large (up to 16 kb), new, smaller, synthetic UCOEs that still lead to high expression of the transgene have been engineered (40,41). Most of the currently used UCOEs derive from the HNRPA2B1-CBX3 *locus*, a genomic region encompassing the promoters of the heterogeneous nuclear ribonucleoprotein A2/B1 (HNRPA2B1) and of the chromobox protein homologue 3 (CBX3) housekeeping genes.

In this study, we used a dual-reporter system to evaluate epigenetic regulatory elements for their ability to protect transgenes expression and to modify the chromatin structure when placed in the prevailing heterochromatic environment of telomeres. Two fluorescent protein-coding reporter genes were placed in a telomeric-proximal or telomeric-distal position relative to a regulatory element, and the strength of various epigenetic regulators was assessed in terms of barrier activity, anti-silencing effect and general transcriptional activation capability. We show that epigenetic regulators such as MAR, UCOE and the chicken cHS4 insulator resulted in an increased number of cells expressing the telomeric distal reporter gene, whereas the telomeric-proximal reporter gene was usually highly repressed, implying a barrier effect, while some of the MAR elements showed an additional anti-silencing activity. Chromatin immunoprecipitation (ChIP) on monoclonal cell populations revealed that epigenetic regulators act differently on the chromatin structure. The positive effects on transgene expression of MAR elements may be due to the enrichment of both histones H3 and H4 acetylation in their proximity, whereas UCOE may prevent the deposition of heterochromatic histone marks such as H3K9me3, H4K20me3 and H3K27me3. Overall, we conclude that the MAR and UCOE strong chromatin boundary as well as the MAR anti-silencing activity may result from the deposition of distinct chromatin structures.

## MATERIALS AND METHODS

### Plasmids and constructs

**Figure 1.**
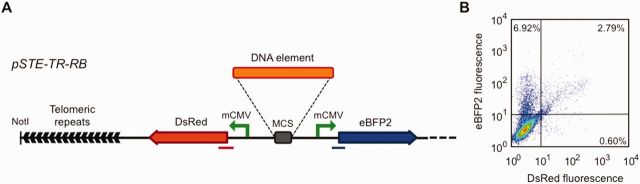
A dual reporter system for the study of epigenetic regulators at human telomeres. (**A**) Features of the reporter constructs used to assess the capacity of epigenetic regulatory elements in protecting gene expression at telomeres. Note the telomeric-proximal and telomeric-distal position of DsRed and eBFP2 relative to the MCS used to insert distinct regulatory or spacer control elements (DNA element). Quantitative PCR amplicons for both DsRed and eBFP2 are shown as bars underneath their respective location. (**B**) Expression profile of stably transfected HeLa cells carrying pSTE-TR-RB shows preferential silencing of the telomeric-proximal gene and moderate expression level of the telomeric-distal eBFP2 in a minority of cells.

A novel dual reporter system for the study of epigenetic regulators at human telomeres was used. Construction of this new system, termed pSTE-TR-RB, started with the introduction of a new multiple cloning site (MCS) into pBS-Puro, a BlueScript vector containing a puromycin resistance cassette. DsRed and eBFP2 (enhanced blue fluorescent protein 2) coding sequences under the control of minimal CMV (mCMV) promoter were polymerase chain reaction (PCR) amplified from pCMV-DsRed Express (Clontech) and pEBFP2 (Addgene Plasmid number 14 893 devoid of the nuclear localization signal) respectively. These sequences, together with telomeric repeats from pGE2min plasmid (42), were cloned into pBS-Pure giving the final construction. Thus, pSTE-TR-RB contains a stretch of telomeric repeats for *de novo* telomere formation at the genomic integration site, a MCS for the insertion of DNA elements, and two distinct reporter genes, DsRed and eBFP2 being respectively at a telomeric proximal and telomeric distal position relative to the MCS (Figure 1A). All DNA elements were cloned between the two reporter genes. Human MAR elements 1-68, X-29 and murine MAR S4 have been previously described (30,34), as were the STAR 7 and 40 elements (36). The multimerized core of MAR X-29 and the core of MAR 1-68 with flanking regions were a kind gift of Dr Salina Arope (Arope *et al.,* manuscript in preparation). The UCOE corresponds to the BspEi-Esp3I fragment of the HNRPA2B1-CBX3 *locus* (41), while the chicken β-globin insulator (cHS4) was taken from the pJC5-4 plasmid kindly provided by Dr Gary Felsenfeld (42). Neutral DNA sequences of different sizes (0.5 kb, 1 kb, 2 kb, 3 kb and 5.6 kb) originated from the PCR amplification of the murine utrophin coding sequence (GenBank accession number: BC062163.1) were also cloned between eBFP2 and DsRed as spacer controls (see also the Supplementary Data).

### Cell culture and transfections

HeLa Tet-off cells (Clontech) were cultivated at 37°C, 5.5% CO_2_ in DMEM:F12 medium (Gibco) supplemented with 10% foetal bovine serum (Gibco). Low passage HeLa cells were transfected with linearized plasmids using FuGENE6 according to the manufacturer’s instruction (Roche). Selection was carried out with 2.5 μg/ml puromycin (Sigma) for 15 days, after which selection was stopped and cells were grown for at least 2 weeks without selection pressure.

#### Flow cytometry

Expression of reporter genes in transfected HeLa cells was measured by a CyAn ADP cell analyzer (Dako) and analysed with FlowJo software (Treestar). Sorting of cells for the establishment of monoclonal populations was done with a FACSAriaII (BD Biosciences) at the Flow Cytometry Core Facility of the Swiss Institute of Technology of Lausanne (EPFL, Switzerland).

### ChIP

HeLa cells were cross-linked in 1% formaldehyde for 7 min and lysed in the presence of protease inhibitor cocktail (Complete tablets, Roche). Chromatin was then sonicated on ice until sheared to an average size of 400 bp, diluted in ChIP dilution buffer [20 mM 4-(2-Hydroxyethyl)piperazine-1-ethanesulfonic acid (HEPES), 200 mM NaCl, 0.1% sodium deoxycholate, 1% Triton X-100, protease inhibitors] and pre-cleared for 90 min in the presence of rProtein A sepharose beads (GE Healthcare Life Sciences). The pre-cleared chromatin was immunoprecipitated overnight at 4°C. Antibodies against acetylated histone H3 (06-599), acetylated histone H4 (06-866) and trimethylated histone H3K27 (07-449) were obtained from Millipore, whereas antibodies against trimethylated histone H3K4 (ab8580), monomethylated H2BK5 (ab12929), monomethylated H3R2 (ab15584), trimethylated histone H3K9 (ab8898) and trimethylated histone H4K20 (ab9053) were purchased from Abcam. Anti GFP antibody was used as negative control (Roche). The Immunoprecipitated complexes were recovered by incubation with 12 μl of rProtein A sepharose beads followed by four washes with immunoprecipitation buffer [20 mM HEPES, 200 mM NaCl, 2 mM ethylenediaminetetraacetic acid (EDTA), 0.1% sodium deoxycholate, 1% Triton X-100]. Beads were re-suspended in elution buffer [100 mM Tris–HCL, 1% sodium dodecyl sulphate (SDS)] and reverse cross-linked overnight at 65°C. Precipitated DNA was recovered using the Qiagen PCR purification kit.

### Quantitative PCR

The quantification of ChIP DNA recovery was performed by quantitative PCR using a LightCycler 480 instrument with SYBR Green-reagent (both from Roche). For the amplification of eBFP2 and DsRed, a combination of a gene-specific forward primer with a common reverse primer for both DsRed and eBFP2 on the mCMV promoter was used. Primer sequences were as follows: eBFP2 forward primer 5′-CAGCTCCTCGCCCTTGCTCA-3′, DsRed forward primer 5′-CGCACCTTGAAGCGCATGAA-3′, mCMV promoter reverse primer 5′-AGAGCTGGTTTAGTGAACCGTCAGATC-3′. The relative positions of these amplicons are shown in Figure 1A. The enrichment of various histone modifications, was calculated for both reporter genes as follows:

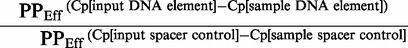

where PP_Eff_ corresponds to the primer pair efficiency calculated using LinRegPCR software (43).

## RESULTS

### Specific regulatory elements increase the number of expressing cells

Different epigenetic regulators, including MARs, UCOE, STARs and other elements previously shown to positively influence transgene expression (Table 1), were cloned into pSTE-TR-RB, a novel molecular reporter system for the study of epigenetic regulators at human telomeres (Figure 1A, see also the Supplementary Data). In addition to epigenetic regulators, neutral sequences of various lengths were also cloned as spacer controls, as the strength of telomeric silencing in HeLa cells was found to partly depend on the distance from the telomeres (*see* Figure S2 of the Supplementary Data), similarly to what was observed in yeast (12). Stable transfection of pSTE-TR-RB in the absence of any DNA element resulted in the silencing of the telomeric-proximal reporter gene (DsRed), whereas the telomeric-distal eBFP2 was expressed by few cells (Figure 1B). This dual reporter system was designed to assess two distinct molecular effects, namely a chromatin boundary activity and a transcriptional activator function. In yeast, heterochromatin propagates from the telomere towards the centromere and a similar mechanism is believed to occur also in higher eukaryotes. Thus, a strong insulator would lead to a higher number of cells expressing the telomeric-distal eBFP2 reporter gene, while a powerful transcriptional activator would rather increase expression levels of both reporter genes, including the telomeric-proximal DsRed.

**Table 1.** Epigenetic regulators and control elements used in this study

DNA elements	Element size (kb)	Plasmid full name	Short name
Empty vector	–	pSTE-TR-RB	0 kb
0.5 kb Utrophin control fragment	0.5	pSTE-TR-RB-0.5 kb	0.5 kb CTRL
1 kb Utrophin control fragment	1	pSTE-TR-RB-1 kb	1 kb CTRL
2 kb Utrophin control fragment	2	pSTE-TR-RB-2 kb	2 kb CTRL
3 kb Utrophin control fragment	3	pSTE-TR-RB-3 kb	3 kb CTRL
5.6 kb Utrophin control fragment	5.6	pSTE-TR-RB-5.6 kb	5.6 kb CTRL
Human MAR 1-68	3.6	pSTE-TR-RB-1-68	1-68
Human MAR X-29	3.5	pSTE-TR-RB-X-29	X-29
Murine MAR S4	5.4	pSTE-TR-RB-S4	S4
Chicken Lysozyme MAR	3	pSTE-TR-RB-Lys	c-Lys
Human MAR 1-68 Core + flanking region	1.4	pSTE-TR-RB-C1-68	Core 1-68
4X Core MAR X29	0.8	pSTE-TR-RB-CX29	Core X-29
Chicken beta-globin HS4 Insulator	1.2	pSTE-TR-RB-HS4	cHS4
UCOE from the HNRPA2B1-CBX3 locus	1	pSTE-TR-RB-UCOE	UCOE
STAR Element 7	2.1	pSTE-TR-RB-STAR7	STAR 7
STAR Element 40	1	pSTE-TR-RB-STAR40	STAR 40

**Figure 2.**
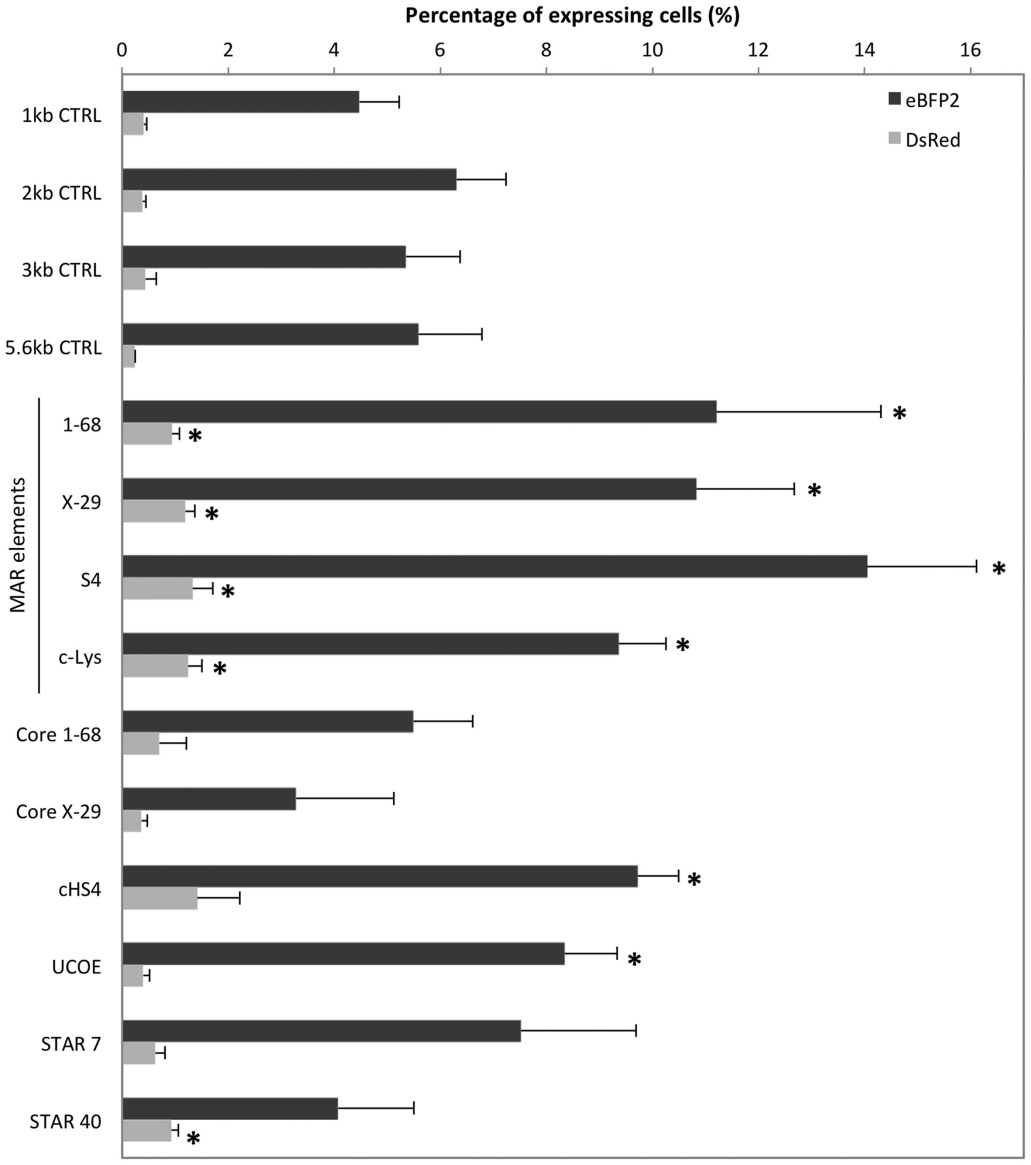
Effect of epigenetic regulators on expressing cell occurrence in polyclonal populations. HeLa cells were stably transfected with pSTE-TR-RB vector bearing different epigenetic regulators or control sequences under antibiotic selection. After 2 weeks of further culture without selective pressure, cells were analysed by flow cytometry and the percentage of cells expressing either reporter gene was assessed in each cell population. Mean and standard deviation of three independent transfections are shown. Stars indicate statistically significant increase in the number of expressing cells compared with control populations where DsRed and eBFP2 are separated by neutral DNA sequences of equivalent size (Student’s *t*-test, *P < 0.05).

Analysis of polyclonal populations of stably transfected cells revealed that most of the DNA elements were able to increase significantly the number of cells expressing at least one of the two reporter genes when compared to a plasmid containing a neutral control sequence of similar length (Figure 2). In stable populations carrying full-length MAR elements or the cHS4 insulator, the number of cells expressing eBFP2 approximately doubled when compared to the control populations. To a lesser extent, the UCOE also led to an increase of eBFP2-positive cells, suggesting a barrier activity for MARs as well as for the cHS4 insulator and UCOE. Together with STAR 40, but to a greater extent, MARs also increased the number of DsRed-positive cells. However, while eBFP2 was expressed in a large number of cells (>5%), the number of DsRed-positive cells in both the control and experimental populations remained generally lower, <1%, but it was nevertheless statistically significant (Figure 2 and Supplementary Figure S1), suggesting an anti-silencing effect for these elements when placed at a telomeric location.

To confirm that DsRed silencing was due to telomeric integration, plasmids depleted of telomeric repeats were constructed for several of these elements. These internal controls showed an overall higher number of expressing cells. Importantly, the occurrence of expressing cells was similar for both eBFP2 and DsRed genes, as expected from a non-telomeric random genomic integration of the reporter cassette (Supplementary Figure S1, pSTE-RB-HS4 and pSTE-RB-S4 constructs). Overall, these results showed a significant barrier activity for MAR and UCOEs, as well as for the previously known cHS4 insulator. In addition, a transcriptional activating effect was observed for MARs and, to a lesser extent, for STAR 40. Interestingly, the AT-rich core sequence of MAR 1-68 and MAR X-29 were unable to mediate a barrier or transcriptional effect, implying that other elements are required to mediate the MAR effect, in agreement with previous bioinformatics-based modelling of MAR elements (30).

### Epigenetic regulatory elements mediate defined expression pattern in monoclonal populations

**Figure 3.**
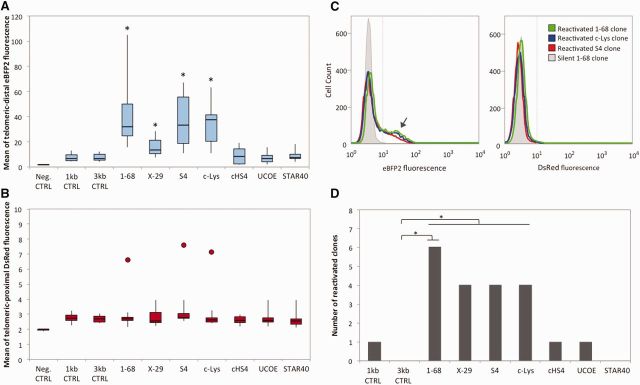
Analysis of monoclonal populations. Stably transfected and expressing cells were sorted into single cells and resulting monoclonal populations grown without selective pressure. For each epigenetic regulator, 12 expressing clones were analysed by cytofluorometry to determine the mean fluorescence of both eBFP2 and DsRed. Mean fluorescence of individual clones are represented as box plots of eBFP2 (**A**) and DsRed (**B**) and they were compared to that of cells generated with the spacer control of equivalent size (Student’s *t*-test: *P < 0.05; *n* = 12). Dots represent outlier clones which were excluded from further analysis. Mean fluorescence in untransfected HeLa cells is also shown as reference (Neg CTRL). Additionally, silenced cells (expressing neither eBFP2 nor DsRed) from stably transfected polyclonal populations were also sorted as monoclonal populations and further grown in the absence of selective pressure. Transgene expression in these silenced populations was followed by FACS over a period of 2 weeks (**C**), after which some clones displayed detectable eBFP2 fluorescence (indicated by an arrow). (**D**) Number of clones displaying eBFP2 expression activation events occurring within the silenced monoclonal populations, showing a significantly higher frequency for MAR elements taken together and for MAR 1-68 relative to the 3 kb spacer control (Fisher's exact test, *P < 0.05; *n* = 24).

Stably transfected cells carrying an epigenetic regulator that decreased the telomeric silencing effect in polyclonal populations (i.e. the full MAR, UCOE, cHS4 and STAR 40 elements), together with their respective spacer controls (1 kb and 3 kb CTRLs), were sorted into monoclonal populations and grown without selection pressure. Clones displaying homogeneous and relatively low expression of DsRed were retained, in order to exclude cells with potential non-telomeric integration of multiple plasmid copies. The mean fluorescence of eBFP2 and DsRed was assessed using 12 clones generated for each of the DNA element and spacer control by flow cytometry, to evaluate the expression variability between distinct clones (Figure 3A and B). The spacer control clones (1 kb and 3 kb CTRLs) displayed little variation and had a generally very low eBFP2 and DsRed expression. Despite a higher variability, an overall significant increase in eBFP2 fluorescence was observed for clones generated with the MARs, whereas other DNA elements did not augment eBFP2 expression. On the other hand, none of the epigenetic regulators yielded significant DsRed expression, which remained at extremely low levels, except for three clones generated with human MAR 1-68, mouse MAR S4 and the chicken lysozyme MAR elements (Figure 3B). Thus, these MAR elements may also act as transcriptional activators, even in the strongly repressive chromatin environment of telomeres.

Starting from the stably transfected polyclonal populations, non-expressing cells were also sorted and grown as monoclonal populations. Although no expression could be detected by fluorescence-activated cell sorting (FACS), PCR amplification on genomic DNA extracted from some silenced cloned confirmed that the reporter genes were still integrated into the genome (data not shown), implying a strong silencing effect rather than a loss of the integrated vector. Among these silent clonal populations, several were able to spontaneously recover eBFP2-expressing cells after 2 weeks of culture without selection (Figure 3C). The number of clones that recovered expression was assessed for each DNA element by scoring populations that displayed more than 1% of expressing cells. MAR 1-68 was the only element which yielded statistically significant reactivation of the silenced eBFP2 transgene, with 6 out of 24 clones that displayed eBFP2-positive cells after 2 weeks of culture without selection (Figure 3D). Nonetheless, all MAR elements yielded clones that displayed reactivated expression, and, taken as a group, they significantly increased the probability of reactivation events. Other elements also yielded occasional reactivating clones, but the frequency was similar to that observed for the control spacer fragments. Overall, MAR 1-68 was the most potent element in terms of actively switching nearby genes from a silent to an expressing state.

### Effects of epigenetic regulatory elements on telomeric chromatin modifications

In order to shed light on the mechanism of action of the epigenetic regulatory elements used in this study, the epigenetic landscape on the reporter genes promoter was assessed by ChIP. Different histone modifications were investigated, including open chromatin marks such as histone 3 and histone 4 acetylation, trimethylated Histone H3 on lysine 4 (H3K4me3), monomethylated H2B on lysine 5 (H2BK5me1) and monomethylated H3 on arginine 2 (H3R2me1), as well as repressive/heterochromatic marks such as trimethylated H3K27 (H3K27me3), trimethylated H3K9 (H3K9me3) and trimethylated H4K20 (H4K20me3).

**Figure 4.**
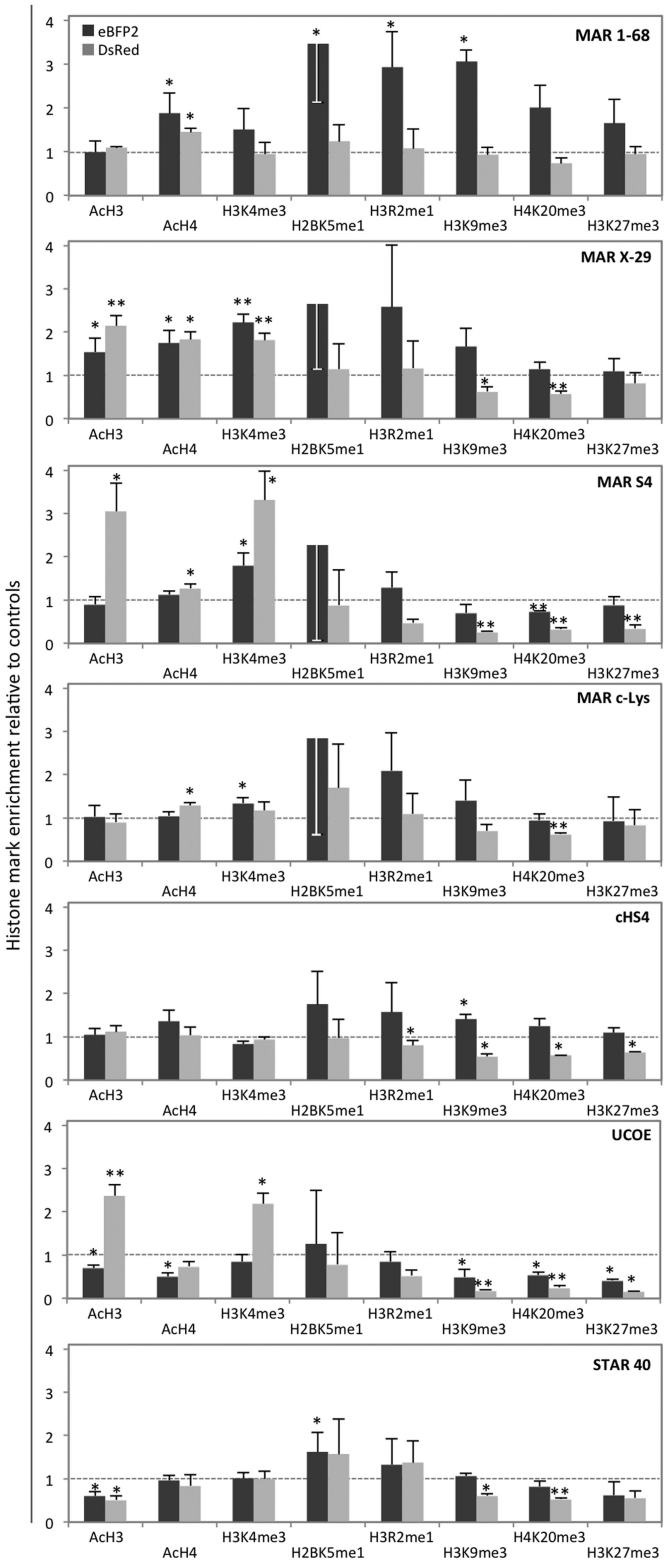
Effect of epigenetic regulatory elements on histone modifications. The presence of histone modifications on eBFP2 and DsRed transgenes was assessed by ChIP with antibodies against acetylated H3 and H4, monomethylated H2BK5 and H3R2 and trimethylated H3K4, H3K9, H4K20 and H3K27. Immunoprecipitated DNA was analysed by quantitative PCR. For each DNA element, two clones were analysed and the mean and standard deviation of three independent experiments are represented as fold enrichment relative to the spacer control of similar size (Student’s *t*-test, *P < 0.05; **P < 0.01).

For each DNA element and its respective spacer control, two representative clones were studied by ChIP. The expression profile of the analysed clones was determined at the time of chromatin preparation (Supplementary Figure S2), and it was shown to correlate well with the mRNA levels of the transgenes (Supplementary Figure S3). Fluorescence *in-situ* hybridization (FISH) analysis was performed on metaphasic chromosomal spreads using probes consisting of the telomeric repeats and of the dual reporter system without DNA elements nor telomeric repeats (pSTE-RB), which confirmed a single integration site at telomeric/subtelomeric position for all analysed clones (Supplementary Figure S4). The copy number of integrated transgenes per genome was also estimated by qPCR on total cellular DNA, yielding values indicative of single copy integration (data not shown). For every genetic element, enrichment values from ChIP experiments performed on the two clones were averaged, to minimize possible clone-specific effects, and they were expressed as fold increase over the values obtained from the controls with spacer DNA of similar size, as obtained from three independent ChIP experiments performed with at least two different chromatin preparations (Figure 4).

Analysis of the absolute histone modification levels for the spacer control clones revealed an enrichment of marks previously associated with (sub)telomeric regions such as H3K9me3, H4K20me3, H2BK5me1 or H3K2me1 on the promoter of the telomeric-proximal reporter gene, as expected from the *de novo* telomere formation at the site of the transgene integration (Supplementary Figure S5). At the chromatin level, the human MAR 1-68 and X-29 and the murine MAR S4 appeared to be the most active DNA elements in terms of augmenting the chromatin marks associated with a permissive chromatin structure (Figure 4 and Supplementary Figure S5). The MAR X-29 promoted deposition of active chromatin marks on both reporter genes, with an increase in the acetylation of histone H3 (AcH3) and H4 (AcH4) and the trimethylation of H3K4 compared with the spacer control. In addition, it reduced the presence of heterochromatic histone marks on the telomere proximal gene (H3K9me3 and H4K20me3). MAR S4 acted mainly on the telomere proximal gene, increasing the deposition of H3K4me3 and acetylated H3 and H4, as well as decreasing the level of all three repressive/heterochromatic marks. MAR 1-68 acted mostly on the telomeric distal gene, which is the gene whose expression could be activated from a silent state (Figure 3 and Supplementary Figure S3). Expression of eBFP2 in the presence of MAR 1-68 was found to be accompanied with increased levels of euchromatic marks, such as AcH4, H2BK5me1 and H3R2me1, as well as with increased levels of H3K9me3, a modification often associated with heterochromatin. In presence of the chicken lysozyme MAR, little differences were detected for active and inactive chromatin modifications, possibly reflecting the fact that it was found to be a relatively weaker MAR element when compared to the human and murine MARs (30). Nonetheless, a significant increase in AcH4 was observed also for this MAR on the telomeric-proximal gene, making the increase of the acetylation of histone 4 the only modification shared by all the MAR elements tested here (Figure 4).

The analysed UCOE was also particularly active at the chromatin level. Besides a strong depletion of all repressive marks—with H3K9me3 and H3K27me3 levels below or close to the background obtained with immunoprecipitation controls—UCOE strongly promoted acetylation of H3 and H3K4 trimethylation over the promoter of the telomere-proximal DsRed gene (Figure 4). Yet, this did not suffice to activate DsRed expression, unlike MAR S4 which had a comparable epigenetic profile (Figure 2 and Supplementary Figure S2). The cHS4 insulator resulted in lower levels of heterochromatic marks on the telomeric-proximal reporter gene, which, together with the absence of effects on euchromatic marks, is consistent with the barrier function proposed for this element. Presence of STAR 40 led to a reduction of both eu- and heterochromatic marks, which might explain the lack of strong effects on gene expression. Overall, ChIP results showed epigenetic landscapes which varied from element to element, with a tendency for MARs to promote the deposition of active histone marks, whereas the UCOE prevented deposition of repressive modifications, as summarized in Table 2.

**Table 2.** Histone modifications and other transcriptional activities associated with epigenetic regulators

DNA element	Boundary activity^a^	Transcriptional activation^b^	Reactivation^c^	Histone modifications^d^							
				Euchromatin			(Sub)telomeric		Heterochromatin		
				AcH3	AcH4	H3K4me3	H2BK5me1	H3R2me1	H3K9me3	H4K20me3	H3K27me3
Human MAR 1-68	**+ +**	**+ +**	**+ +**	**∼**	**+**	**∼**	**∼**	**∼**	**∼**	**∼**	**∼**
				**∼**	**+**	**∼**	**+ +**	**+ +**	**+ +**	**∼**	**∼**
Human MAR X-29	**+ +**	**+**	**+**	**+ +**	**+**	**+**	**∼**	**∼**	**−**	**−**	**∼**
				**+**	**+**	**+**	**∼**	**∼**	**∼**	**∼**	**∼**
Murine MAR S4	**+ +**	**+ +**	**+**	**+ +**	**+**	**+ +**	**∼**	**∼**	**− −**	**− −**	**− −**
				**∼**	**∼**	**+**	**∼**	**∼**	**∼**	**−**	**∼**
Chicken Lysozyme MAR	**+**	**+ +**	**+**	**∼**	**+**	**∼**	**∼**	**∼**	**∼**	**−**	**∼**
				**∼**	**∼**	**+**	**∼**	**∼**	**∼**	**∼**	**∼**
HS4 Insulator	**+ +**	**−**	**−**	**∼**	**∼**	**∼**	**∼**	**−**	**−**	**−**	**−**
				**∼**	**∼**	**∼**	**∼**	**∼**	**+**	**∼**	**∼**
1 kb UCOE Element	**+**	**−**	**−**	**+ +**	**∼**	**+ +**	**∼**	**∼**	**− −**	**− −**	**− −**
				**−**	**−**	**∼**	**∼**	**∼**	**− −**	**−**	**− −**
Star Element 40	**−**	**−**	**−**	**−**	**∼**	**∼**	**∼**	**∼**	**−**	**−**	**∼**
				**−**	**∼**	**∼**	**+**	**∼**	**∼**	**∼**	**∼**

^a^The boundary or barrier activity is shown for each element, as determined by the number of eBFP2 expressing cells in polyclonal populations (Figure 2). Signs are based on the fold increase of expressing cells for a given element relative to cells transfected with the control construct with a spacer DNA of similar size. ++, + and – signs represent a >2 fold, a ≤2 fold but still significant, and a non-significant increase of eBFP2-positive cells, respectively.

^b^The median cell fluorescence of expressing clones was used to determine transcriptional activation (Figure 3A). Significant fold increases of >3 (++) or >2 (+) fold are shown.

^c^The frequency of reactivation events, based on the number of silent clones which display expressing cells after 2 weeks of culture (Figure 3D) are shown as ++ (>20%), + (>10%) or – (≤10%) for each epigenetic element.

^d^Enrichment (+) and depletion (−) of the different histone marks on either the telomeric-proximal DsRed (grey signs) or the telomeric-distal eBFP2 (black signs) based on ChIP data (Figure 4). Double signs correspond to a fold increase >2-fold (++) or <0.5-fold (−−) relative to the spacer control. A tilde sign (∼) indicates that no significant change was observed relative to the spacer control.

## DISCUSSION

The eukaryotic genome is functionally compartmentalized into defined chromatin domains that are thought to contribute to the regulation of different biological processes including gene transcription. Integration of a transgene in the host genome may result in inconsistent expression, as elicited by the particular chromatin environment of the integration site. Epigenetic regulators are DNA sequences which may minimize this position effect, possibly leading to a more consistent expression of the transgene (22). Here, we tested different epigenetic DNA elements for their ability to protect transgene expression from the silencing mediated by the strongly unfavourable chromatin environment resulting from a telomeric location.

We show that MARs can act to protect gene expression at telomeric *loci*. MARs greatly increased the proportion of cells expressing a telomeric-distal reporter gene, while they less frequently prevented the silencing of a telomeric-proximal transgene, suggesting a prominent barrier activity for this class of epigenetic regulators. In addition, MAR elements could reactivate expression from silent clones at a high frequency, and thus possess anti-silencing effects. Finally, MARs also increased the transcription rate of the telomere-distal gene, as assessed from the fluorescence and mRNA levels, indicating that they can also act as transcriptional activators. These conclusions are further supported by the finding that the MARs locally promoted modifications of the chromatin to adopt a structure more permissive for gene expression. In particular, acetylation of histone H4 was enriched in proximity of all four MAR elements tested, while further histone H3 acetylation was promoted by the two potent MARs X-29 and S4. The presence of acetylated nucleosomes may result from the recruitment of histone acetyltransferases, as proposed for other MAR elements in human osteosarcoma U2OS cell line (44). Interestingly, histone deacetylation was reported to be among the first steps towards TPE in human cells, and it is essential for the successive histone methylations required for the establishment of telomeric heterochromatin (19). This suggests that MARs may counteract the propagation of silent chromatin by imposing acetylation marks on adjacent nucleosomes, thereby acting as insulator or barrier elements. In addition, the murine MAR S4 and the human MAR X-29 also promoted the deposition of trimethylated H3K4, which is usually mapped on active promoters (45) and partially prevented histones from being decorated with trimethylated H3K9 and H4K20, two heterochromatic marks (46). This correlates well with the strong initial increase of expression mediated by these two MARs (30,34).

The effects of some MARs on transgene expression was limited in this study by the single copy integration upon *de novo* telomere formation at the integration site. Indeed, the increased transgene expression resulting by the insertion of MARs is in part also due to a higher number of integrated copies (47,48). This could partially explain the comparable impact of the human MAR 1-68 on chromatin modifications and gene expression, when assessed in parallel with MAR S4 and MAR X-29, despite the fact that the 1-68 element is one of the strongest MAR characterized so far in stable transfection studies (30,34). Nevertheless, we found that clones carrying the MAR 1-68 had a high and stable expression of eBFP2 in spite of the enrichment of H3K9me3 on the eBFP2 promoter. Thus, MAR 1-68 might be able to force transcription despite the presence of H3K9me3 on the promoter, or, as suggested by previous studies, H3K9me3 may also be associated to actively expressed promoters in particular genomic contexts (49–51). Additionally, the MAR 1-68 was found to confer high stability to gene expression upon prolonged culture time (35), and we show here that it significantly increases the activation rate of silent telomeric genes, which correlates well with the long-term sustainment of transgene expression. Overall, these results may explain the enhanced and stable transgene expression previously observed in the presence of MAR elements such as this MAR upon integration at internal chromosomal sites (31).

UCOEs are CpG island-containing DNA sequences derived from the promoter regions of housekeeping genes (38), and the relatively short element used here was shown to retain most of the characteristics of larger UCOE-containing sequences (41). At telomeric *loci*, the UCOE could increase the number of cells expressing the telomeric distal reporter gene, as indicative of protection against silencing effects by a barrier effect, but it did not increase fluorescence levels as would result from higher transcription rates. Interestingly, the chromatin environment in proximity to the UCOE was poor in histone modifications, particularly in heterochromatic repressive marks. One possible explanation is that UCOE are nucleosome-poor regions, in agreement with the findings that CpG islands are relatively nucleosome-deficient (52) or that there is a relative lack of nucleosomes at the endogenous promoters of the HNRPA2B1-CBX3 UCOE *locus* (38). Concerning the lack of expression of the DsRed gene despite the UCOE-mediated deprivation of heterochromatic marks, we speculate that additional euchromatic modifications such as the hyperacetylation of histone H4 may be required for gene transcription, as observed in the presence of MAR elements, while UCOE rather opposed H4 acetylation. Overall, and despite different experimental designs, our findings are in agreement with data showing that expression at the endogenous HNRPA2B1-CBX3 UCOE *locus* was accompanied by active histone modification marks such H3K4me3 and acetylation of both histones H3 and H4, together with low repressive marks such as H3K27me3 (38). The lack of such a pattern in the presence of UCOE at telomeric *loci* may thus explain the lack of silent gene activation.

The chicken beta-globin HS4 element is a well-known insulator that was shown to protect transgene expression from TPE when the transgene was insulated by two copies of cHS4 (53). Here we showed that a single copy of the cHS4 insulator suffices to protect a telomeric-distal gene expression from TPE. Together with MAR elements, the chicken insulator cHS4 is the element which gave the highest percentage of eBFP2 positive cells, consistently with a strong boundary activity. Similarly to MAR 1-68, we did detect some H3K9me3 enrichment on the promoter of eBFP2, in agreement with a prior study of HS4 by Rincon-Arano *et al.* (52). Previous studies at non-telomeric *loci* showed that the barrier activity of cHS4 was accompanied by acetylation of nearby histones (54,55). However, we did not find histones near cHS4 to be highly acetylated, possibly reflecting the fact that in our construction transgenes were not flanked by cHS4, but that a single copy of cHS4 was present. Different models have been proposed for the mechanisms of barrier action. Some predict the active recruitment of histone-modifying enzyme such as histone acetyltransferases, whereas some are purely based on spatial interference to the heterochromatic spread, possibly by DNA looping mechanisms (56). Our data therefore support the notion that the cHS4 insulator may function differently depending on the chromatin environment present at the integration site.

STAR elements were proposed to prevent the deposition of heterochromatin-related proteins such as heterochromatin protein 1 (36). At telomeres, STARs were thus expected to act as barriers, preventing the spread of heterochromatin. However, in our study, STARs had overall weaker or unclear effects. In polyclonal populations, STARs gave contrasting results, with STAR element 7 being inefficient in protecting either of the two reporter genes from silencing, while STAR 40 increased only the number of cells expressing the telomere-proximal DsRed gene. ChIP experiments revealed that STAR 40 reduced the levels of heterochromatic marks H3K9me3 and H4K20me3 on the telomere proximal gene only, supporting a role as an anti-silencing element. However, this anti-repressor activity of STAR40 was not sufficient to promote high levels of protection from TPE, and neither STAR element had an effect on transcription rate at the telomeric *loci*.

Over 10 years ago, it was proposed that histone modifications are not isolated chromatin marks, but that they constitute together a histone code, as represented by the combinatorial patterns of histone modifications that function in concert (57). These patterns of histone modifications have been observed across the genome of many species (58), and they are also evidenced in our study, as distinct patterns of chromatin marks were elicited by the various epigenetic regulators studied. Overall, data presented here also indicate that the presence of a single mark of open chromatin, or the absence of a repressive histone mark, cannot be solely correlated to gene expression level or resistance to silencing. Consistently with the histone code proposal, our data rather indicate that multiple combinations of chromatin modifications regulate gene expression transitions, and they may pave the way towards a more rational use of such epigenetic regulatory DNA elements and histone modifications to achieve more predictable transgene expression patterns.

## SUPPLEMENTARY DATA

Supplementary Data are available at NAR Online, including [59–71].

## FUNDING

Swiss Commission for Technology and Innovation and from Selexis SA, and by the University of Lausanne. Funding for open access charge: University of Lausanne.

*Conflict of interest statement*. N.M. is a co-founder and own shares of Selexis SA, a company that uses proprietary technology to generate therapeutic-producing CHO cell lines. Other authors have no competing interest to declare.

## Supplementary Material

Supplementary Data
