# Supplementary material for: Epigenetic regulatory elements associate with specific histone modifications to prevent silencing of telomeric genes
Source: Nucleic Acids Res. 2013 Sep 25;42(1):193–204. doi: 10.1093/nar/gkt880 (PMC3874193; doi:10.1093/nar/gkt880)
Supplement: Supplementary Data [file supp_42_1_193__index.html]

Epigenetic regulatory elements associate with specific histone modifications to prevent silencing of telomeric genes — Supplementary Data 

# Epigenetic regulatory elements associate with specific histone modifications to prevent silencing of telomeric genes

## Supplementary Data

files

**Files in this Data Supplement:**

- Supplementary Data - pdf file
- Supplementary Data - pdf file
- Supplementary Data - pdf file
