# Supplementary material for: Epigenetic regulatory elements associate with specific histone modifications to prevent silencing of telomeric genes
Source: Nucleic Acids Res. 2013 Sep 25;42(1):193–204. doi: 10.1093/nar/gkt880 (PMC3874193; doi:10.1093/nar/gkt880)
Supplement: Supplementary Data [file supp_gkt880_MajocchiEtAl-SupplFigs.pdf]

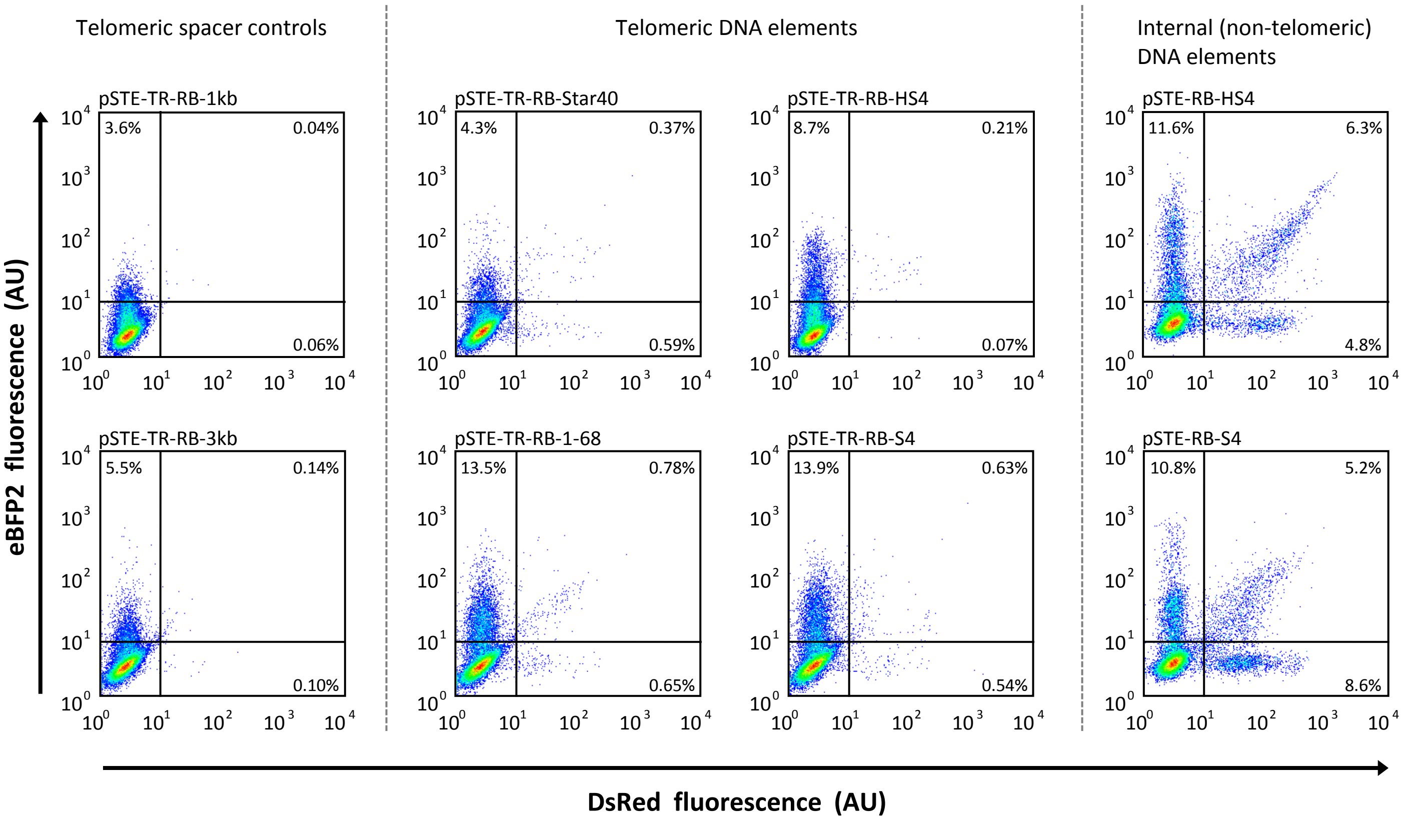

Supplementary Figure 1 – Reporter genes expression in polyclonal populations. Representative expression profiles of HeLa cells stably transfected with vector bearing spacer controls of different size (left panel), various DNA elements integrated either at telomeric *loci* (central panel) or at random internal chromosomal position (right panel). Cells were grown for two weeks without selective pressure before being analyzed by flow cytometry.

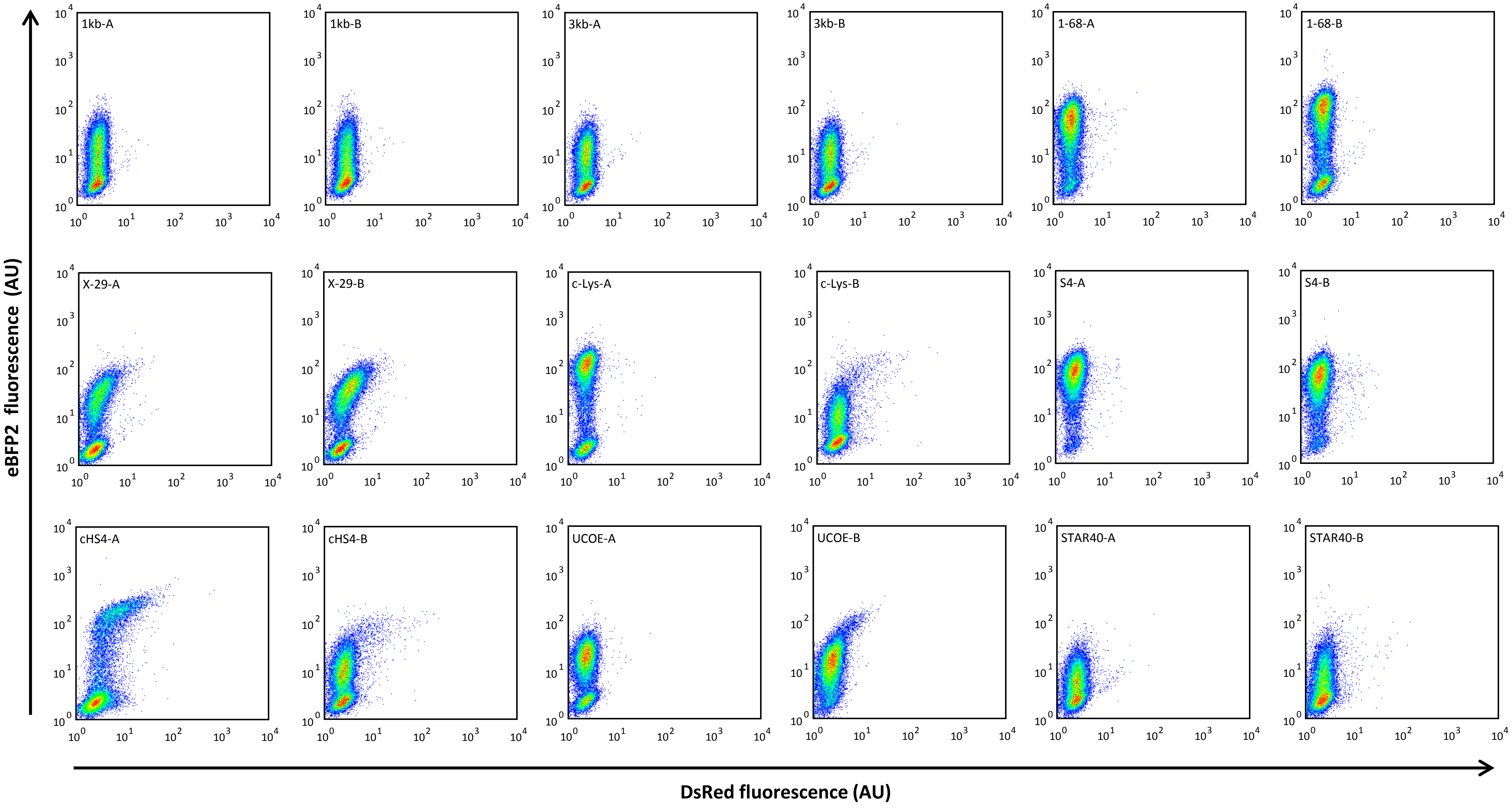

Supplementary Figure 2 – Reporter genes expression in monoclonal populations.
Two representative clones for each DNA element (including spacer controls) were retained for chromatin immunoprecipitation (ChIP) experiments. Reporter gene expression levels in retained clones were assessed by FACS. Analysis representative of clone expression pattern at the time of chromatin preparation for ChIP experiments are shown.

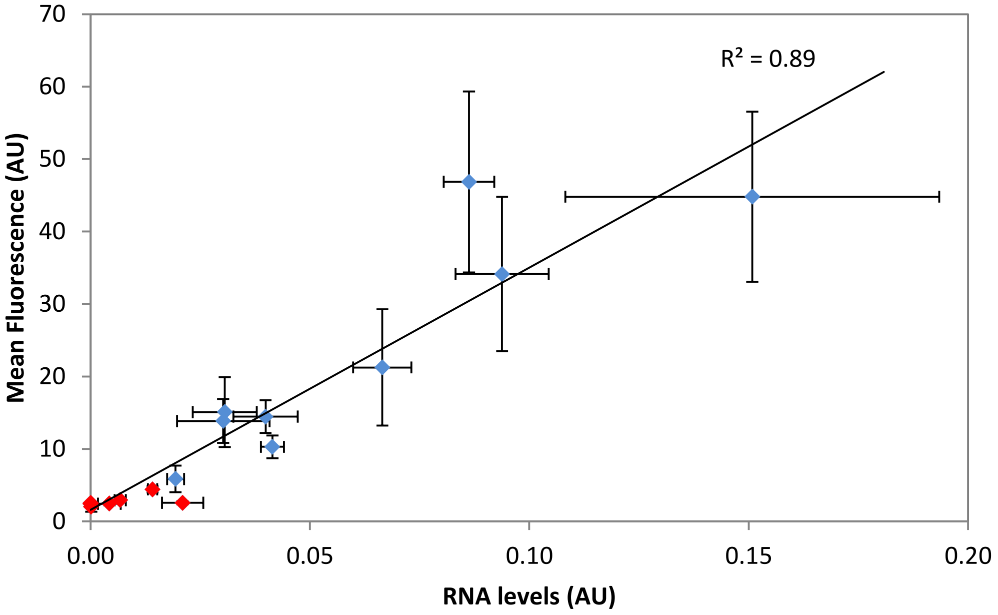

Supplementary Figure 3 – Correlation between RNA and fluorescence levels in monoclonal populations.

RNA levels were quantified by qRT-PCR and compared to fluorescence level as determined by FACS [see supplementary methods]. A correlation between RNA and fluorescence levels is observed, indicating that FACS read-out is representative of the transcription levels within monoclonal populations. DsRed and eBFP2 measurements are represented as red and blue diamonds, respectively.

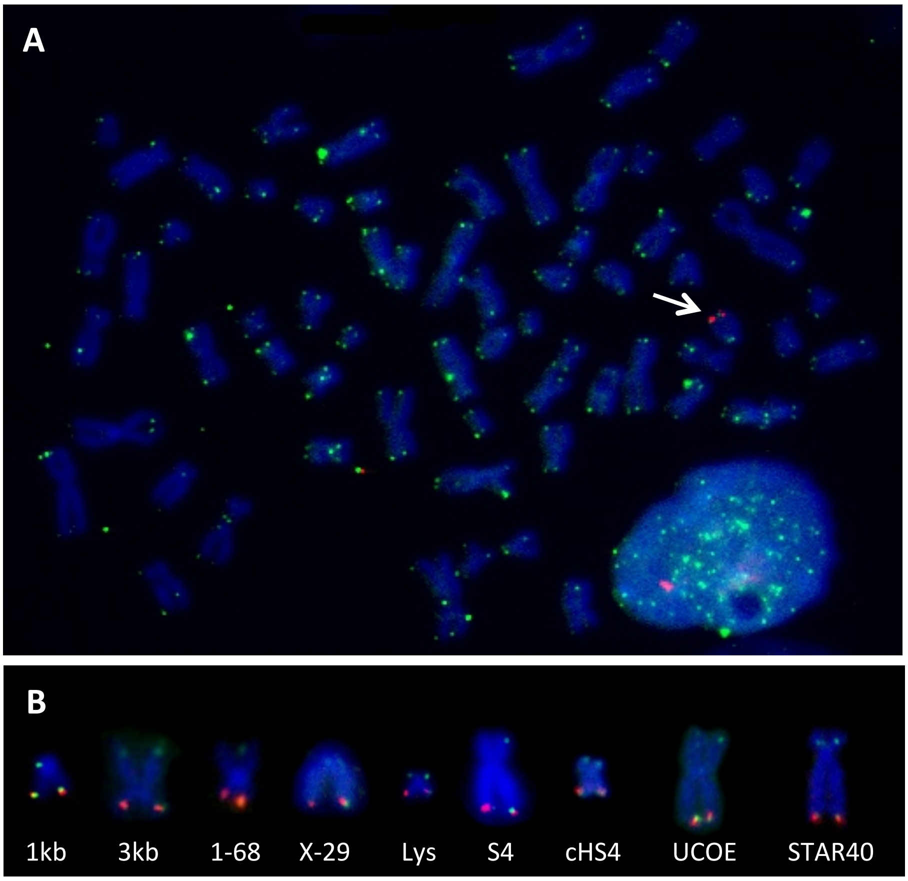

Supplementary Figure 4 – Localization of transgene integration in monoclonal populations.

(A) Telomeric integration was assessed by FISH with probes targeting the integrated vector (red) and telomeric repeats (green) [see supplementary method]. Chromosomes were counterstained with DAPI. Arrow points at the location of the transgene integration site.

(B) *In situ* hybridization was performed for all clones. Retained clones had integration at telomeric *loci*, albeit on different chromosomes.

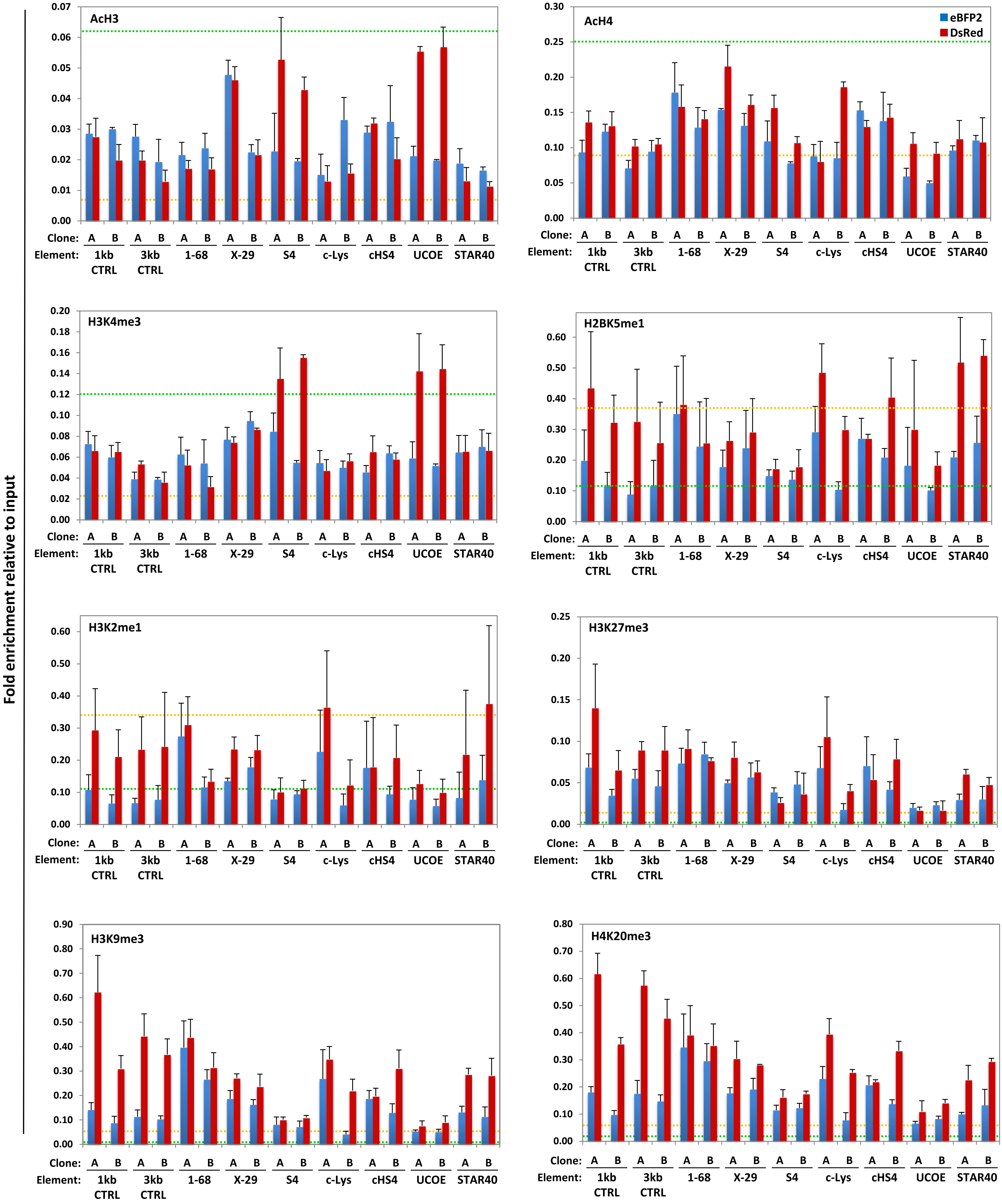

Supplementary Figure 5 – Chromatin Immunoprecipitation (ChIP) raw data

The promoter regions of both reporter genes were investigated for association with selected histone modifications by chromatin immunoprecipitation. Two different clones (A and B) were analyzed for every regulatory element. Mean and SD of three independent experiments on at least two different chromatin preparations are shown. Mean enrichments of GAPDH (green dotted line) and Serpina1 (orange dotted line), two endogenous genes being respectively actively transcribed or inactive in most human tissue, are also shown as reference.
