# Supplementary material for: Epigenetic regulatory elements associate with specific histone modifications to prevent silencing of telomeric genes
Source: Nucleic Acids Res. 2013 Sep 25;42(1):193–204. doi: 10.1093/nar/gkt880 (PMC3874193; doi:10.1093/nar/gkt880)
Supplement: Supplementary Data [file supp_gkt880_nar-01531-f-2013-File008.pdf]

Majocchi et al., SUPPLEMENTARY DATASET AND REFERENCES:

A new molecular tool for the study of epigenetic regulators at human telomeres

INTRODUCTION

Transgene expression highly depends on the integration site of the coding sequence, as the local chromatin environment and/or the presence of regulatory elements can deeply affect the transcription rate of a gene of interest (GOI). This is a problem not only for academic research, where the GOI may not be expressed at sufficient level in cell lines or in transgene animals, but also for industrial recombinant protein production. DNA elements capable of reducing position effects and resulting in an increased expression of the transgene independently of the integration site may be used (22). Few studies aiming at comparing such epigenetic regulators have been performed (59,60). However, comparisons were generally performed following random integration at distinct internal chromosomal sites, where the particular integration spot may deeply influence the outcome of the study and thus made any comparison less reliable. Targeting various expression vectors to telomeres, where each vector contains a different DNA element may allow comparison of these epigenetic regulators in more defined chromatin settings, minimizing the effect of different integration sites.

Telomeres have been considered genomic regions highly unfavourable for gene expression, mainly because of their prevailing heterochromatic structure. In both lower and higher eukaryotes, it is believed that heterochromatin spreads from the telomeres toward the centromeric regions resulting in the silencing of genes located near telomeres (12-15). This phenomenon, named telomere position effect, was exploited for the study of gene expression or silencing, boundary proteins and anti-silencing elements in different organisms. Yeast was the first organism where such studies were conducted, since homologous recombination is highly efficient and allowed precise targeting of reporter genes in proximity of yeast telomeres (12). For mammalian cells, targeting the integration at telomeres was achieved using a linear plasmid containing a terminal stretch of telomeric repeats: upon transfection into human, telomerase-positive cell lines, the linearized plasmid is believed to undergo an illegitimate recombination with the host genome at a random interstitial site, rather than integration at an existing telomere (61-63). Recognition of the repeats as a telomere seed leads then to a *de novo* formation of a functional telomere (62). The use of telomeric repeat to target transgene integration at telomeric *loci*, even though artificial, has been successfully used to study telomeric position effect and/or elements preventing telomeric silencing in mammalian cells (13,14,53).

Nevertheless, these studies relied on a single reporter gene, excluding the possibility to differentiate between a protected telomeric-distal site and a silenced telomeric-proximal one. A dual-reporter system avoiding these limitations was firstly used in yeast, where two divergent reporter genes integrated near the telomeres were used to study the boundary activity of CTF1, the CCAAT-box binding transcription factor 1 (64,65). Besides an N-terminal DNA binding domain, CTF1 contains a proline-rich, C-terminal transactivation domain which was shown to interact with histone H3 (66). Using the above-mentioned dual-reporter system, the proline-rich domain fused to the bacterial Gal4 DNA binding domain (DBD) was shown to act as a boundary element capable of preventing the spread of the silencing signal which originates at telomeres in both yeast and human cells, protecting the expression of gene located downstream – but not upstream – of the Gal-Pro fusion binding site (64,67). Additionally, evidence of boundary activity of the human CTF proteins at mammalian telomeres has also been produced (67). However, these earlier vectors were not ideal to assess unambiguously the relative transcription rates, as the fluorescent markers used were not easily distinguishable by cytofluorometry using common Fluorescence-activated cell sorter (FACS) equipment. Based on the knowledge gained with such earlier studies, we present here a novel dual reporter system for the assessment of epigenetic regulatory elements at human telomeres, where DNA elements can be challenged in a strongly silencing environment for their ability to protect and enhance gene expression.

RESULTS

Construction of a dual reporter system for the study of epigenetic regulators at telomeric loci

A dual reporter system was set up to assess the silencing of transgenes integrated at telomeric loci in the presence of DNA regulatory elements (Figure 1A). Preliminary work was performed to assess which fluorescent reporter proteins might be more easily discriminated by cytofluorometry, which led to the choice of DsRed and eBFP2 (SM and NM, unpublished results). The coding sequences of DsRed and eBFP2 were thus cloned in divergent orientations on either side of a multiple cloning site under the control of minimal cytomegalovirus (mCMV) promoters (Figure 1A). An antibiotic resistance cassette was added at the 3' of the eBFP2 coding sequence for selection purposes, whereas a stretch of human telomeric repeats was inserted at the 3' of the DsRed reporter gene. Transfection of a linear plasmid containing terminal stretch of telomeric repeats into telomerase-positive cells causes *de novo* telomere seeding and formation of a new artificial telomere at the integration site (61,62). This mechanism was exploited in several studies to target transgene integration at telomeric *loci* (13,53,67). The formation of a new telomere at the 3' of the DsRed coding sequence led to a dramatic decrease of DsRed expressors in stably transfected HeLa cells, suggesting a strong silencing effect due to the presence of telomeric repeats (Figure 1B, pSTE-TR-RB). When the relative position of the reporter genes was exchanged, eBFP2, now at telomeric-proximal position, was strongly silenced, indicating that the silencing is position dependent rather than reporter-gene dependent, and that neither of these genes is preferentially silenced or expressed (Figure 1B, pSTE-TR-BR). As control, a plasmid lacking the repeats was also transfected into HeLa cells, showing an overall higher number of expressing cells, more equally distributed DsRed and eBFP2 positive cells, and the frequent occurrence of doubly positive cells, as expected from an internal chromosomal integration (Figure 1B, pSTE-RB).

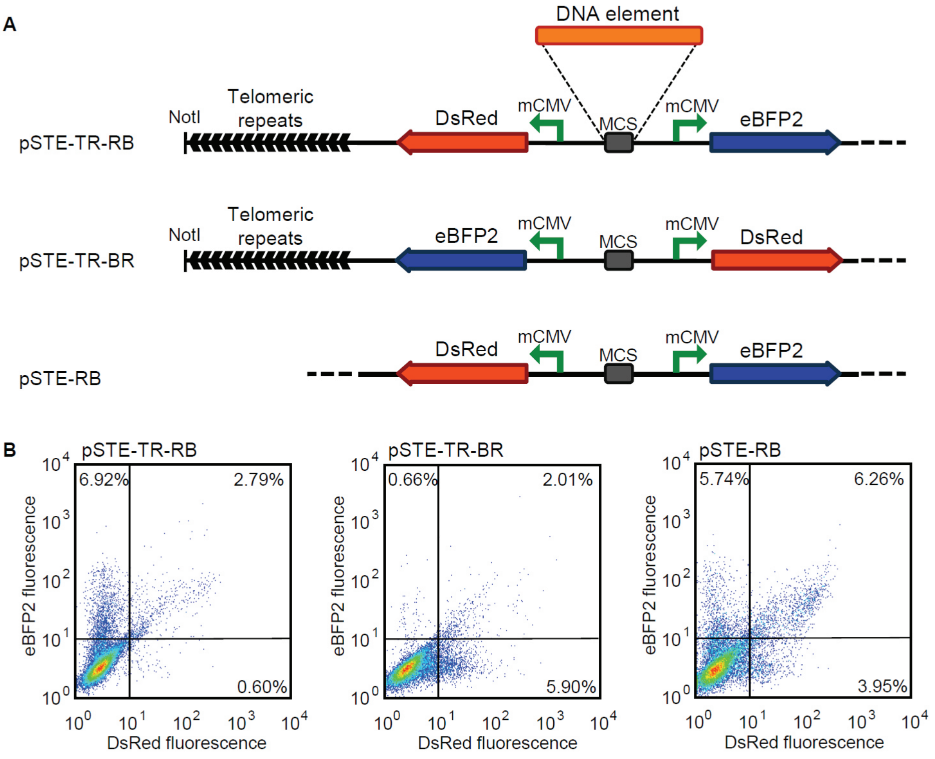

Figure 1. A dual reporter system to study epigenetic regulators at human telomeres.

(A) Key features of pSTE-TR-RB, the vector designed to assess the ability of epigenetic regulators to protect gene expression from telomeric silencing. DsRed and eBFP2 were respectively at a telomeric-proximal and telomeric-distal position of a multiple cloning site (MCS) which may be used to insert epigenetic regulators (DNA element). Both reporter genes were under the control of minimal CMV (mCMV) promoters. Additionally, as controls, a vector where the relative position of DsRed and eBFP2 was reversed (pSTE-TR-BR) as well as a vector devoid of telomeric repeats (pSTE-RB) were generated. (B) Expression profiles of stably transfected HeLa cells carrying pSTE-TR-RB (left panel); pSTE-TR-BR (middle panel), and pSTE-RB (right panel) showing distinct expression patterns reflecting transgene orientation and the presence or absence of telomeric repeats.

Effects of spacer controls on reporter genes expression

Contrary to yeast, where the likelihood of TPE was shown to decrease with the distance from the telomere (12), the characteristics of mammalian TPE remain largely unknown. Therefore, neutral DNA sequences of various lengths were cloned between DsRed and eBFP2, moving the two promoters apart. To minimize the risk of using sequences containing factitious regulatory elements that might affect the expression of the reporter genes, the coding sequence of murine utrophin was used as template for the amplification of spacer controls. Sizes ranging from 0.5kb to 5.6kb were used. Reporter genes expression in stably transfected cells carrying the control plasmids was analysed by FACS (Figure 2A) and the percentage of positive cells was plotted for both DsRed and eBFP2 expression from the different constructs (Figure 2B).

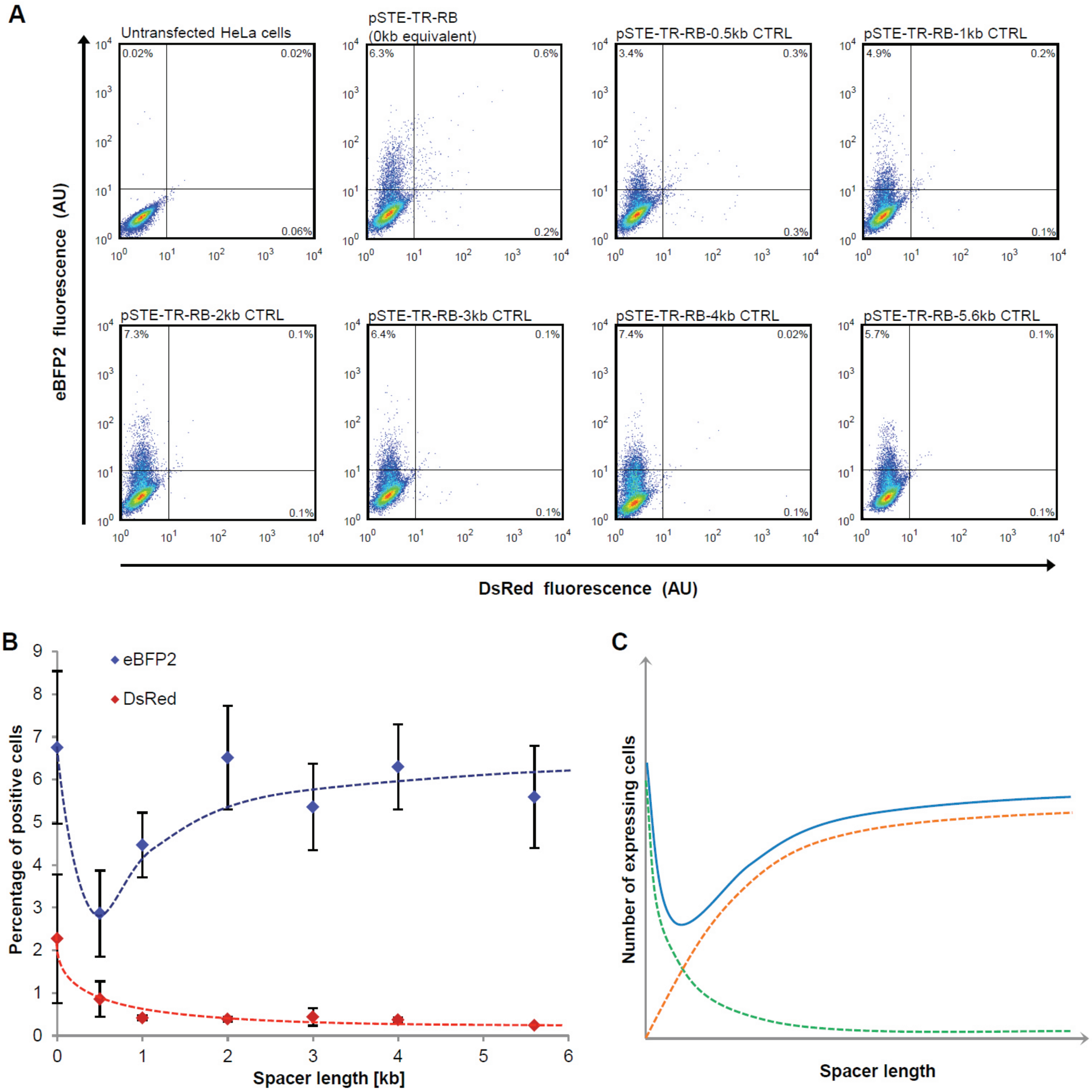

Figure 2. Telomere position effect is distance dependent in human cells.

(A) Representative FACS analysis of stably transfected HeLa cells carrying the different spacer control plasmids after two weeks of selection followed by two weeks without selective pressure. (B) Percentage of expressing cells corresponding to various spacer lengths, depicted as the mean and standard deviation of 3 independent experiments. (C) Model to explain the overall eBFP2 expression in spacer controls. The number of eBFP2 expressing cells is expected to increase with larger spacer controls, similar to what observed in yeast (yellow dotted line). However, a local synergistic effect is observed when the promoters are kept at short distance, resulting in an increase of expressing cells (green dotted line). The sum of these two independent phenomena may explain the empirically observed eBFP2 expression pattern as shown in 2B (blue line).

The number of eBFP2 expressing cells depended on the distance from the telomere, as the lowest number of expressors was observed in the presence of the shortest spacer control (0.5kb). Longer spacer controls resulted in increasing number of eBFP2 positive cells. Despite of the change in the number of eBFP2 expressing cells within the different spacer controls, the intensity of blue fluorescence for all the constructions was approximately constant (data not shown). On the other hand, DsRed expression was deeply repressed independently of the spacer length, most probably because of the unaltered close proximity of the telomeric repeats (Figure 2A). Taken together, these results suggested that TPE strength in HeLa cells depends on the distance from the telomere, similarly to what observed in yeast (12). However, surprising results were obtained with the empty vector, which may be considered as a 0kb spacer control, as some expression of DsRed occurred. When averages of independent experiments were plotted as a relation to the spacer length, expression from the DsRed promoter was found to be influenced by the distance to the eBFP2 promoter, as these are adjacent in the 0kb spacer control. Thus, a synergistic activation between the two minimal promoters may occur when placed at really short distance, which might cause the highest percentage of expressing cells as observed with this construct. Closely located head-to-head promoters were shown to result in more stable gene expression (68). In our case, this promoter configuration might similarly lead to synergistic action of the promoter-binding transcription factors, thereby blocking the spread of telomeric silencing and increasing the number of expressing cells. As a consequence, two contrasting effects have to be considered when analysing gene transcription using a dual-reporter system as depicted on figure 2C, where the orange line shows the increase in expression with increasing distance to the telomere, the green line illustrates the decrease of expression upon the loss of transcriptional synergism when the two promoters are moved further apart, and the blue line show the resulting expression profile as observed experimentally in Figure 2B. As a last step, we validated the system by inserting two well-known epigenetic regulatory elements into pSTE-TR-RB, namely the chicken HS4 (cHS4) insulator (28) and the chicken lysozyme MAR antisilencing element (33). Results shown in Figure 3 below, and presented in more details in the Results section of the main manuscript, illustrate the use of this dual-reporter system to characterize DNA elements in term of three distinct activities, namely a unidirectional barrier effect, an anti-silencing activity exerted on the telomere proximal gene, and the transcriptional activation of either reporter genes.

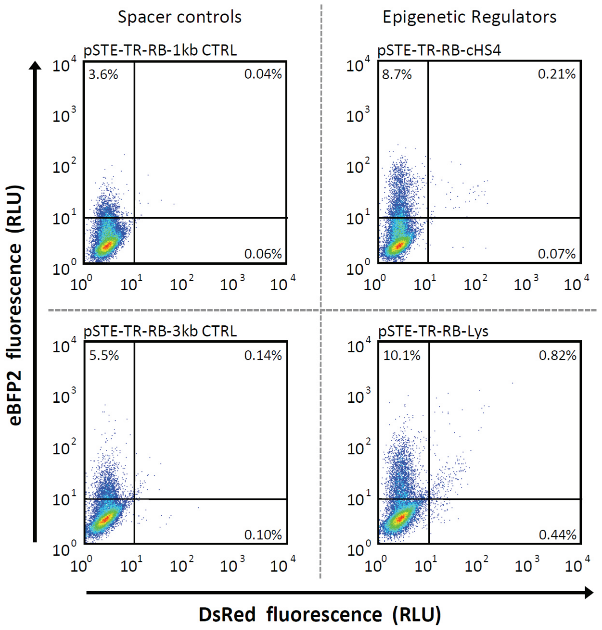

Figure 3 Effect of known epigenetic regulators on reporter genes expression.

Representative expression profiles of polyclonal populations of HeLa cells stably transfected with vector bearing spacer controls of different size (left panel) or known epigenetic regulators (right panel). The 1kb-long chicken HS4 insulator and the 3kb-long chicken lysozyme MAR should respectively be compared to the 1kb and 3kb spacer controls. Cells were grown for two weeks without selective pressure before being analysed by flow cytometry.

DISCUSSION

We designed a new and powerful vector to assess the ability of known epigenetic regulators to protect and enhance gene expression at telomeres, genomic regions that possess a chromatin environment where heterochromatic features prevail and which was shown to be highly unfavourable for protein coding-gene expression. Telomeres are responsible for a reversible silencing of nearby genes which is termed the telomere position effect. By mean of a dual reporter system containing a stretch of telomeric repeats, we specifically induced *de novo* telomere formation at the integration site, therefore resulting in a telomeric integration of the reporter vector. Telomeric integration resulted in very limited expression of the transgenes from the weak promoters used to drive their expression. More precisely, the telomeric-proximal gene was kept under strong telomeric silencing and was most highly repressed, likely because of its position next to the telomeric repeats. However, the number of cells expressing the telomere-distal gene such as eBFP2 reflected the distance to the telomere, indicating that TPE in HeLa cells is distance dependent, like in yeast (12). Based on the data obtained, it is tempting to assume that at *de novo* telomeres in HeLa cells there is a strong correlation between the proximity to the telomeres and the strength of telomeric silencing during the first few kb, that is up to the 2 kb spacer control. However, with larger spacer DNAs, the weakening of TPE with the distance was less striking, but some silencing effect remained, and the occurrence of silenced cells was detected up to several kb from the telomere, similarly to what was observed in yeast (12). Interestingly, the eBFP2 fluorescence levels did not change dramatically with different spacer controls. This indicated that TPE may not influence the transcription rate, but that it rather acts to control the overall state of a promoter (on or off), as may be expected for heterochromatin-mediated silencing, thus affecting mainly the number of expressing cells.

In cells carrying the empty vector, where both reporter gene promoters are adjacent at a short distance from the telomeric repeats, the number of both eBFP2 and DsRed was unexpectedly high. It is tempting to assume that, when placed next to each other, there is a synergistic activation of the two promoters which is strong enough to keep the promoter chromatin in an open configuration in a subset of the cells, in spite of their location close to a telomere. Prior observations that divergently organized gene pairs result in more stable gene expression (68) and that transcription at one locus can positively affect neighbouring genes (69) led to the hypothesis that on a local scale, up-regulation of one gene may directly affect the histone modifications in its proximity, leading to stabilized chromatin landscape and therefore to sustained and stable expression of both genes (70,71). Our results with the empty vector support this hypothesis, as the number of expressing cells sharply dropped as the two promoters were moved 0.5kb apart. Overall, these results showed that two independent effects may control transgene expression in our dual-reporter system upon telomeric integration. On the one hand, TPE exerts its influence on genes located near the telomere, possibly with decreasing silencing strength for increasing distances from the telomeric repeats; on the other hand, a local synergistic effect is suggested when the promoters are within a given distance, which limits the effect of TPE. This showed the importance of having a series of spacer controls of different length, since constructs containing epigenetic regulators cannot be directly compared to the empty vector.

The usefulness of this system for the characterisation and comparison of known epigenetic regulators is further illustrated and commented in the main manuscript. Furthermore, this dual-reporter system might be used for the screening of new strong activators or powerful insulators, for instance by inserting random genomic DNA fragments in pSTE-TR-RB, and screening stably transfected cells carrying the resulting constructions by flow cytometry. These flow cytometry-sorted cells could be grown as monoclonal population, and the sequence responsible of such an effect be amplified and cloned for further studies. Overall, we thus propose this new dual expression system for both the characterization of known epigenetic regulators in the challenging heterochromatic environment of telomeres and for the screening of new insulators and/or transcriptional activators.

SUPPLEMENTARY REFERENCES

12. Gottschling, D.E., Aparicio, O.M., Billington, B.L. and Zakian, V.A. (1990) Position effect at S. cerevisiae telomeres: reversible repression of Pol II transcription. *Cell*, **63**, 751-762.
13. Baur, J.A., Zou, Y., Shay, J.W. and Wright, W.E. (2001) Telomere position effect in human cells. *Science*, **292**, 2075-2077.
14. Koering, C.E., Pollice, A., Zibella, M.P., Bauwens, S., Puisieux, A., Brunori, M., Brun, C., Martins, L., Sabatier, L., Pulitzer, J.F. and Gilson, E. (2002) Human telomeric position effect is determined by chromosomal context and telomeric chromatin integrity. *EMBO Rep*, **3**, 1055-1061.
15. Pedram, M., Sprung, C.N., Gao, Q., Lo, A.W., Reynolds, G.E. and Murnane, J.P. (2006) Telomere position effect and silencing of transgenes near telomeres in the mouse. *Mol Cell Biol*, **26**, 1865-1878.
22. Kwaks, T.H. and Otte, A.P. (2006) Employing epigenetics to augment the expression of therapeutic proteins in mammalian cells. *Trends Biotechnol*, **24**, 137-142.

28. Recillas-Targa, F., Pikaart, M.J., Burgess-Beusse, B., Bell, A.C., Litt, M.D., West, A.G., Gaszner, M. and Felsenfeld, G. (2002) Position-effect protection and enhancer blocking by the chicken beta-globin insulator are separable activities. *Proc Natl Acad Sci U S A*, **99**, 6883-6888.
33. Girod, P.A., Zahn-Zabal, M. and Mermod, N. (2005) Use of the chicken lysozyme 5' matrix attachment region to generate high producer CHO cell lines. *Biotechnol Bioeng*, **91**, 1-11.
53. Rincon-Arano, H., Furlan-Magaril, M. and Recillas-Targa, F. (2007) Protection against telomeric position effects by the chicken cHS4 beta-globin insulator. *Proc Natl Acad Sci U S A*, **104**, 14044-14049.
59. Otte, A.P., Kwaks, T.H., van Blokland, R.J., Sewalt, R.G., Verhees, J., Klaren, V.N., Siersma, T.K., Korse, H.W., Teunissen, N.C., Botschuijver, S., van Mer, C. and Man, S.Y. (2007) Various expression-augmenting DNA elements benefit from STAR-Select, a novel high stringency selection system for protein expression. *Biotechnol Prog*, **23**, 801-807.
60. Zahn-Zabal, M., Kobr, M., Girod, P.A., Imhof, M., Chatellard, P., de Jesus, M., Wurm, F. and Mermod, N. (2001) Development of stable cell lines for production or regulated expression using matrix attachment regions. *J Biotechnol*, **87**, 29-42.
61. Farr, C., Fantes, J., Goodfellow, P. and Cooke, H. (1991) Functional reintroduction of human telomeres into mammalian cells. *Proc Natl Acad Sci U S A*, **88**, 7006-7010.
62. Hanish, J.P., Yanowitz, J.L. and de Lange, T. (1994) Stringent sequence requirements for the formation of human telomeres. *Proc Natl Acad Sci U S A*, **91**, 8861-8865.
63. Barnett, M.A., Buckle, V.J., Evans, E.P., Porter, A.C., Rout, D., Smith, A.G. and Brown, W.R. (1993) Telomere directed fragmentation of mammalian chromosomes. *Nucleic Acids Res*, **21**, 27-36.
64. Ferrari, S., Simmen, K.C., Dusserre, Y., Muller, K., Fourel, G., Gilson, E. and Mermod, N. (2004) Chromatin domain boundaries delimited by a histone-binding protein in yeast. *The Journal of biological chemistry*, **279**, 55520-55530.
65. Gronostajski, R.M. (2000) Roles of the NFI/CTF gene family in transcription and development. *Gene*, **249**, 31-45.
66. Alevizopoulos, A., Dusserre, Y., Tsai-Pflugfelder, M., von der Weid, T., Wahli, W. and Mermod, N. (1995) A proline-rich TGF-beta-responsive transcriptional activator interacts with histone H3. *Genes Dev*, **9**, 3051-3066.
67. Esnault, G., Majocchi, S., Martinet, D., Besuchet-Schmutz, N., Beckmann, J.S. and Mermod, N. (2009) Transcription factor CTF1 acts as a chromatin domain boundary that shields human telomeric genes from silencing. *Mol Cell Biol*, **29**, 2409-2418.
68. Woo, Y.H. and Li, W.H. (2011) Gene clustering pattern, promoter architecture, and gene expression stability in eukaryotic genomes. *Proc Natl Acad Sci U S A*, **108**, 3306-3311.
69. Ebisuya, M., Yamamoto, T., Nakajima, M. and Nishida, E. (2008) Ripples from neighbouring transcription. *Nature cell biology*, **10**, 1106-1113.
70. Wang, G.Z., Lercher, M.J. and Hurst, L.D. (2011) Transcriptional coupling of neighboring genes and gene expression noise: evidence that gene orientation and noncoding transcripts are modulators of noise. *Genome biology and evolution*, **3**, 320-331.
71. Woo, Y.H. and Li, W.H. (2012) Evolutionary conservation of histone modifications in mammals. *Molecular biology and evolution*, **29**, 1757-1767.
