# Supplementary material for: Epigenetic regulatory elements associate with specific histone modifications to prevent silencing of telomeric genes
Source: Nucleic Acids Res. 2013 Sep 25;42(1):193–204. doi: 10.1093/nar/gkt880 (PMC3874193; doi:10.1093/nar/gkt880)
Supplement: Supplementary Data [file supp_gkt880_nar-01531-f-2013-File009.pdf]

Majocchi et al., SUPPLEMENTARY METHODS

Fluorescence *In Situ* Hybridization

Dividing HeLa cells were exposed for 90 minutes to colcemid (Invitrogen) at a final concentration of 0.1 µg/ml to block cell division in metaphase. After harvesting, cells were exposed to a hypotonic shock with 37.5 mM KCl for 20 minutes, fixed with 25% acetic acid and 75% methanol and spread onto superfrost microscope slides. Hybridization probes were prepared using a nick translation DNA labeling system (Enzo Life Sciences) and fluorescent uridine nucleotides according to the manufacturer's instructions. The telomeric probe, marked with Green 496 dUTP (Enzo Life Sciences), was generated from purified telomeric repeats obtained from pSTE-TR-RB. The probe targeting the insert, marked with Orange 552 dUTP (Enzo Life Sciences), was generated from pSTE-RB, a vector deprived of telomeric repeats. After precipitation in ethanol with 50-fold excess of sheared salmon sperm DNA, the probe was resuspended in hybridization buffer (2x SSC, 50% formamide, 10% Dextran Sulfate), denaturated for 10 min at 75°C, cooled down on ice and finally pre-warmed at 37°C. Before applying the probes, slides were washed in PBS for 5 min at room temperature, denaturated for 5 minutes in denaturation buffer (2x SSC, 70% formamide at 75°C), dehydrated through ethanol series performed at room temperature (70%, 85%, 100%; 1 min each) and air-dried. Hybridization occurred overnight at 37°C. Slides were first washed for 90 seconds with 0.4x SSC and 0.3% NP-40 at 72°C followed by a 1 minute wash in 2x SSC and 0.1% NP-40 at room temperature. Metaphases were counterstained with Vectashield Mounting Medium with DAPI (Vector Labs) and observed using a 100X oil immersion objective on an Axio Observer Inverted microscope (Carl Zeiss).

RNA isolation and quantitative reverse transcription PCR (qRT-PCR)

Total RNA was isolated using the TRIzol reagent (Invitrogen) following manufacture's protocol, and consecutively treated with DNase I (NEB) to remove traces of DNA contamination. For qRT-PCR analysis, cDNA was prepared from 1ug of total RNA using oligo-dT primer and the First-Strand cDNA Synthesis Kit (GE Healthcare). The cDNA was amplified using the LightCycler 480 SYBR Green I Master mix and the LightCycler 480 instrument (Roche). For PCR amplification of eBFP2 cDNA, forward and reverse primers were: 5'-CCC AGT CCG TGC TGA GCA AAG A -3' and 5'- TCC GGA CTT GTA CAG CTC GTC CA -3' respectively. DsRed cDNA was amplified with 5'- TGG TGA CCG TGA CCC AGG ACT C -3' forward and 5'- CAG CCC ATA GTC TTC TTC TGC ATT AC-3' reverse primers. eBFP2 and DsRed expression was analysed relative to b-actin expression using the advance relative quantification analysis from the LightCycler 480 Software (version 1.5.0 SP4). b-actin cDNA was amplified with: 5'- TCC TTC CTG GGC ATG GAG -3' forward and 5'- AGG AGG AGC AAT GAT CTT GAT CTT -3' reverse primers. Primer pair PCR efficiency was measured using the LinReg PCR software (linregpcr.nl version 11.0).
